# Longer sleep duration and the incidence and progression of diabetic nephropathy: a prospective cohort study

**DOI:** 10.1210/jendso/bvag201

**Published:** 2026-10-08

**Authors:** Fuki Ikeda, Tomoya Mita, Yusuke Osonoi, Yuki Someya, Takeshi Osonoi, Miyoko Saito, Shiho Nakayama, Hidenori Ishida, Ryota Ishii, Masahiko Gosho, Hirotaka Watada

**Affiliations:** Department of Metabolism & Endocrinology, Juntendo University Graduate School of Medicine, Bunkyo-ku, Tokyo 113-8421, Japan; Department of Metabolism & Endocrinology, Juntendo University Graduate School of Medicine, Bunkyo-ku, Tokyo 113-8421, Japan; Department of Metabolism & Endocrinology, Juntendo University Graduate School of Medicine, Bunkyo-ku, Tokyo 113-8421, Japan; Department of Metabolism & Endocrinology, Juntendo University Graduate School of Medicine, Bunkyo-ku, Tokyo 113-8421, Japan; Naka Kinen Clinic, Naka City, Ibaraki 311-0113, Japan; Naka Kinen Clinic, Naka City, Ibaraki 311-0113, Japan; Department of Metabolism & Endocrinology, Juntendo University Graduate School of Medicine, Bunkyo-ku, Tokyo 113-8421, Japan; Naka Kinen Clinic, Naka City, Ibaraki 311-0113, Japan; Department of Biostatistics, Institute of Medicine, University of Tsukuba, Tsukuba, Ibaraki 305-8575, Japan; Department of Biostatistics, Institute of Medicine, University of Tsukuba, Tsukuba, Ibaraki 305-8575, Japan; Department of Metabolism & Endocrinology, Juntendo University Graduate School of Medicine, Bunkyo-ku, Tokyo 113-8421, Japan

**Keywords:** sleep, diabetic nephropathy, lifestyle habits, metabolic profile

## Abstract

**Background:**

Sleep duration has been linked to adverse health outcomes, but its association with diabetic nephropathy remains unclear.

**Objective:**

This study aimed to assess the association between sleep duration and a composite endpoint of incidence and progression of diabetic nephropathy.

**Methods:**

The study participants were 678 Japanese outpatients with type 2 diabetes. Lifestyle habits were assessed at baseline and at years 2 and/or 5. Mean values of lifestyle factors were calculated by averaging measurements from baseline to the date of endpoint onset or the end of follow-up. A multivariable Cox proportional hazards model was used.

**Results:**

During a follow-up of 6.0 ± 2.7 years, 215 participants experienced the endpoint. Among lifestyle factors, mean sleep duration was the only factor associated with the endpoint. Compared with participants sleeping <6 hours, the multivariable-adjusted hazard ratios for mean sleep duration of 6 to <7, 7 to <8, and ≥8 hours were 1.08 (95% CI, 0.74-1.57), 1.56 (95% CI, 1.05-2.31), and 1.92 (95% CI, 1.15-3.19), respectively (*P* for trend = .003). This association remained significant after adjustment for key lifestyle and diabetes-related factors. The association was stronger in older participants and those with microalbuminuria or reduced kidney function.

**Conclusion:**

Longer, but not shorter, sleep duration is associated with an increased risk of incidence and progression of diabetic nephropathy.

Diabetic nephropathy (DN) is among the most prevalent and severe complications of diabetes, affecting approximately 30% to 40% of people with the condition (1). It is strongly associated with the development of end-stage renal disease and an increased risk of cardiovascular events (1).

Previous studies have suggested that various lifestyle factors contribute to kidney function decline. While the associations between dietary protein intake and changes in estimated glomerular filtration rate (eGFR) or urinary albumin excretion (UAE) have been inconsistent among people with reduced baseline kidney function or those with type 2 diabetes without macroalbuminuria (2-4), other lifestyle factors such as salt intake, physical activity, smoking, and alcohol consumption have also been reported to be associated with kidney function decline and an increased risk of proteinuria (5, 6).

In recent years, the widespread use of the Internet and smartphones, along with the globalization of activities, has established a 24-hour society in which individuals are active around the clock. Given that many people experience disrupted sleep-wake cycles and insomnia, both short and long sleep durations have been reported to be associated with various adverse health outcomes, including obesity and weight gain (7), diabetes (8), hypertension (9), cardiovascular disease (10), chronic kidney disease, and mortality (11) in the general population. Among people with type 2 diabetes, the American Diabetes Association guidelines also indicate that both short and long sleep durations are associated with elevated HbA1c levels (11). Furthermore, cross-sectional studies have reported that both short and long sleep durations are associated with decreased eGFR and increased albuminuria in people with type 2 diabetes (12, 13). In addition to sleep duration, recent cohort studies have suggested that poor sleep quality may be associated with an increased risk of kidney function decline in people with type 2 diabetes (14). However, the associations between sleep habits and the incidence and progression of DN in people with type 2 diabetes have not been fully elucidated.

Accordingly, this study aimed to assess the associations of lifestyle habits, including sleep habits, with the incidence and progression of DN in people with type 2 diabetes.

## Materials and methods

### Study participants

Participants were enrolled from outpatient clinics at 3 medical institutions. The study design and inclusion and exclusion criteria were previously published (15-17). The inclusion criteria were (1) a diagnosis of type 2 diabetes, (2) ≥25 and <70 years of age, and (3) provision of written informed consent for study participation. The exclusion criteria were as follows: (1) type 1 or secondary diabetes; (2) severe infectious disease or severe trauma before or after surgery; (3) history of myocardial infarction, angina pectoris, cerebral stroke, or cerebral infarction; (4) chronic renal failure requiring hemodialysis; (5) liver cirrhosis; (6) moderate or severe heart failure (New York Heart Association stage III or higher); and (7) active malignancy. Between June 2013 and January 2014, 736 outpatients were recruited for this study. They were observed for approximately 10 years to monitor the incidence of events. Throughout the follow-up period, participants continued to receive standard diabetes treatment from their regular healthcare providers, with no limitations imposed on the types of medications used.

The study protocol was approved by the Ethics Committee of Juntendo University Hospital. This study conformed to the provisions of the Declaration of Helsinki. Written informed consent was obtained from all the participants. This study was registered in the University Hospital Medical Information Network Clinical Trials Registry (UMIN000010932).

### Study outcomes

The outcome was a composite endpoint of the incidence and progression of DN, which had been predefined as one of the secondary outcomes. DN was categorized based on UAE levels: normoalbuminuria was defined as UAE <30 mg/g creatinine, microalbuminuria as 30 to 300 mg/g creatinine, and macroalbuminuria as ≥300 mg/g creatinine (18). The onset of albuminuria was defined as the first occurrence of microalbuminuria or macroalbuminuria in participants with normoalbuminuria at baseline (18), and the progression of microalbuminuria was defined as the first transition from microalbuminuria to macroalbuminuria (18).

### Questionnaire survey

Questionnaire surveys were administered, as previously described (15-17). Participants’ typical meal timing over the month prior to the study was assessed using the validated Brief Self-Administered Diet History Questionnaire (BDHQ). Estimated daily energy intake was calculated with a custom-built computer algorithm that quantified intake from 58 food and beverage items (19). In the BDHQ, protein intake is categorized as either animal- or plant-based (20). Dietary intake of total protein, as well as other major nutrients, was presented as percentages of energy (% energy). Chronotype was determined using the Morningness–Eveningness Questionnaire (MEQ) (21), which yields scores between 16 and 86; lower values indicate an evening-type chronotype, while higher values indicate a morning-type chronotype. Sleep quality was measured using the Pittsburgh Sleep Quality Index (PSQI) (22). Depressive symptoms were assessed using the Beck Depression Inventory-II (23), with higher scores indicating a depressive state. Physical activity levels were evaluated through the International Physical Activity Questionnaire (24).

Chronotype and sleep quality were evaluated at baseline, year 2, and year 5, while assessments of dietary habits, physical activity, and depressive symptoms were conducted at baseline and again at year 2. The participants were asked about their average night sleep duration over the past month at each of the 3 time points. The mean values for lifestyle factors were calculated as the average of baseline and follow-up values, up to the date of event incidence or the end of follow-up, whichever came first. These values could reflect long-term lifestyle habits beyond those measured at baseline. Smoking status was determined by asking participants whether they currently smoked or had done so in the past; individuals were then categorized as never, former, or current smokers.

### Data collection

Blood and urine samples were obtained at baseline and annually thereafter for up to 8 years, as previously described (15-17). UAE was determined using a latex agglutination assay, and eGFR was calculated based on a previously validated equation (25).

### Statistical analyses

Results are presented as means ± standard deviations or medians (quartiles 1 and 3) for continuous variables and as numbers (proportions) of participants for categorical variables. Baseline and follow-up group comparisons were assessed using Student's t-test or Wilcoxon's rank-sum test for continuous variables and with χ^2^ or Fisher's exact test for categorical variables.

To assess the association between each lifestyle habit and the composite endpoint, univariate and multivariable Cox proportional hazards models were used to estimate hazard ratios (HRs) and their 95% confidence intervals (CIs). The main multivariable model included age and sex, plus potential risk factors with a *P* value < .10 in a univariate Cox proportional hazards model. Another multivariable model was used for the endpoint as a sensitivity analysis: Model 1 (crude) and Model 2 (baseline age and sex). Baseline age and sex were included in all multivariable models as prespecified covariates. Additional potential confounding factors were selected based on their associations with the endpoint in univariate analyses (*P* < .10) and were subsequently included in the main multivariable model. Lifestyle variables were modeled as time-averaged exposures using repeated measurements obtained at baseline and follow-up visits (years 2 and/or 5), whereas all other covariates, including medication use, were included as baseline values in the Cox proportional hazards models.

In further analyses, the participants were categorized into approximately 1-hour sleep duration intervals (<6, 6 to <7, 7 to <8, and ≥8 hours). These categories were determined based on the distribution of sleep duration in the present cohort (Fig. S1) (26) while maintaining sufficient numbers of participants in each category and were consistent with those used in previous epidemiological studies (27-29). Associations between these sleep duration categories and the composite endpoint were evaluated using the multivariable Cox proportional hazards model described above. Trend estimation for linear trends across groups was based on linear regression analysis for continuous variables and logistic regression analysis for categorical variables. Both longitudinal trajectories and changes from baseline to each follow-up time point in cardiometabolic parameters across groups were evaluated using trend analyses.

All statistical tests were two-sided, with a significance level of 5%. All analyses were performed using the SAS software version 9.4 (SAS Institute, Cary, NC, USA).

## Results

### Participant characteristics

Among the 736 participants, 678 with available baseline UAE data and at least one follow-up UAE measurement were included in the outcome analyses after excluding those with macroalbuminuria at baseline. During the mean follow-up period of 6.0 ± 2.7 years, 215 (31.7%) participants experienced the composite endpoint.

Table 1 summarizes the baseline characteristics of participants with and without the endpoint (n = 215 and 463, respectively). Participants who met the endpoint were significantly older, had a longer duration of diabetes, higher systolic blood pressure, and exhibited higher levels of HbA1c, triglycerides, and UAE compared with those who did not. The use of sulfonylureas, glucagon-like peptide-1 receptor agonists, and antiplatelet agents was more frequent among participants with the endpoint. No significant differences in lifestyle habits were observed between the two groups.

**Table 1:** Baseline demographics and characteristics of the study cohort, stratified by presence or absence of the composite endpoint of incident and progressive diabetic nephropathy

Parameters	No endpoint (n = 463)	Endpoint (n = 215)	*P* value
Sex (male) (%)	287 (62.0)	133 (62.4)	.910
Age (years)	57.3 ± 8.4 (n = 463)	58.9 ± 8.4 (n = 215)	.025
Body mass index	24.5 ± 4.2 (n = 463)	24.8 ± 3.9 (n = 213)	.380
Duration of diabetes (years)	8.0 (4.0 to 12.0) (n = 463)	10.0 (6.0 to 14.0) (n = 210)	.010
HbA1c (%)	6.87 ± 0.89 (n = 463)	7.07 ± 1.06 (n = 215)	.012
Systolic blood pressure (mmHg)	125.6 ± 13.8 (n = 463)	128.0 ± 14.3 (n = 213)	.042
Diastolic blood pressure (mmHg)	76.8 ± 10.2 (n = 463)	76.4 ± 10.6 (n = 213)	.702
Total cholesterol (mg/dL)	185.4 ± 27.3 (n = 463)	183.4 ± 28.1 (n = 214)	.394
Low-density lipoprotein cholesterol (mg/dL)	104.5 ± 23.9 (n = 462)	101.7 ± 25.6 (n = 215)	.166
High-density lipoprotein cholesterol (mg/dL)	59.5 ± 14.1 (n = 463)	58.3 ± 13.0 (n = 215)	.287
Triglycerides (mg/dL)	98.0 (70.0 to 140.0) (n = 459)	111.0 (72.0 to 159.5) (n = 212)	.015
Uric acid (mg/dL)	5.41 ± 1.23 (n = 463)	5.57 ± 1.24 (n = 215)	.130
Estimated glomerular filtration rate (mL/min/1.73 m^2^)	78.6 ± 16.5 (n = 463)	77.2 ± 19.4 (n = 213)	.335
Urinary albumin excretion (mg/g creatinine)	8.3 (4.8 to 16.5) (n = 463)	14.4 (8.6 to 23.5) (n = 215)	< .001
Presence of diabetic retinopathy	155(33.5)	81(38.6)	.199
Use of glucose-lowering agents			
Metformin (%)	224 (48.4)	115 (54.0)	.175
Sulfonylureas (%)	29 (6.3)	29 (13.6)	.002
Glinides (%)	9 (1.9)	2 (0.9)	.517
Dipeptidyl peptidase-4 inhibitors (%)	157 (33.9)	69 (32.4)	.698
Sodium-glucose cotransporter 2 inhibitors (%)	0 (0)	0 (0)	—
Thiazolidinediones (%)	19 (4.1)	19 (4.2)	1.000
α-Glucosidase inhibitors (%)	263 (56.8)	112 (52.6)	.305
Glucagon-like peptide-1 receptor agonists (%)	22 (4.8)	23 (10.8)	.003
Insulin (%)	40 (8.6)	26 (12.2)	.147
Use of antihypertensive drugs			
Angiotensin-converting enzyme inhibitors	3 (0.6)	1 (0.5)	1.000
Angiotensin II receptor blockers	190 (41.0)	96 (45.1)	.324
Calcium channel blockers	94 (20.3)	57 (26.8)	.061
Use of lipid-lowering agents			
Statins	188 (40.6)	79 (37.1)	.385
Fibrates	23 (5.0)	14 (6.6)	.394
Use of antithrombotic agents			
Antiplatelet agents	10 (2.2)	12 (5.6)	.033
Morningness–Eveningness Questionnaire (points)	57.2 ± 7.0 (n = 463)	57.9 ± 7.8 (n = 214)	.258
Pittsburgh Sleep Quality Index (points)	5.1 ± 2.9 (n = 463)	5.1 ± 3.1 (n = 214)	.955
Beck Depression Inventory-II (points)	9.7 ± 7.3 (n = 463)	9.9 ± 7.9 (n = 215)	.714
Smoker (current/former/never)	101/140/222 (21.8/30.2/47.9)	52/57/106 (24.2/26.5/49.3)	.570
Alcohol consumption (g/d)	0.2 (0.0 to 12.0) (n = 463)	0.3 (0.0 to 23.9) (n = 214)	.192
Total energy intake (kcal/d)	1652.1 (1322.0 to 1992.5) (n = 463)	1661.3 (1345.1 to 2027.1) (n = 214)	.423
Physical activity (Mets/h/wk)	19.5 (7.4 to 47.8) (n = 462)	16.5 (6.5 to 48.0) (n = 214)	.252
Sleep duration (h)	6.4 ± 1.2 (n = 463)	6.5 ± 1.1 (n = 214)	.330

Data are presented as numbers (%), means ± standard deviations, or medians (quartiles 1-3).

### Association between lifestyle habits and the composite endpoint of diabetic nephropathy

We assessed the association between lifestyle habits and the endpoint. In crude Cox proportional hazards models, mean sleep duration, mean total energy intake, the mean proportion of carbohydrate intake, the mean proportion of plant-based protein intake, mean salt intake (g/day), and mean alcohol consumption (g/day) were associated with the incidence of the endpoint (*P* < .10) (Table S1) (26). After adjustment for age and sex, similar associations were observed, except for salt intake (Table S1) (26). Among conventional risk factors, estimated duration of diabetes, HbA1c, systolic blood pressure, log triglycerides, uric acid, log UAE, and the use of sulfonylureas, glucagon-like peptide-1 receptor agonists, calcium channel blockers, and antiplatelet agents at baseline were associated with the endpoint (*P* < .10) (Table S2) (26). In a multivariable model including lifestyle habits and these conventional risk factors, mean sleep duration was the only lifestyle factor that tended to be associated with the risk of the endpoint (HR, 1.13; 95% CI, 0.98 to 1.30; *P* = .082) (Table 2), prompting further analyses focusing on sleep duration.

**Table 2:** Multivariable Cox proportional hazards model for the endpoint

Exploratory variable	HR (95% CI)	*P*
Mean sleep duration (hours)	1.13 (0.98 to 1.30)	.082
Mean alcohol consumption (10 g/day)	1.07 (0.99 to 1.16)	.106
Mean total energy intake (100 kcal/day)	1.01 (0.98 to 1.04)	.523
Mean carbohydrate intake (% energy)	0.99 (0.97 to 1.02)	.620
Mean plant-based protein intake (% energy)	0.98 (0.83 to 1.15)	.808
Age (years)	1.02 (1.00 to 1.04)	.075
Sex (female) vs male	1.25 (0.90 to 1.73)	.191
Duration of diabetes (years)	1.01 (0.99 to 1.03)	.487
HbA1c (%)	1.20 (1.03 to 1.40)	.022
Systolic blood pressure (mmHg)	1.00 (0.99 to 1.01)	.873
Log triglycerides	1.04 (0.80 to 1.36)	.770
Uric acid (mg/dL)	1.12 (0.99 to 1.28)	.071
Log urinary albumin excretion	1.39 (1.21 to 1.58)	<.001
Sulfonylurea (yes)	1.43 (0.95 to 2.15)	.087
Glucagon-like peptide-1 receptor agonists (yes)	1.89 (1.19 to 3.02)	.007
Calcium channel blockers (yes)	1.02 (0.74 to 1.41)	.908
Antiplatelet agents (yes)	1.45 (0.76 to 2.75)	.255

Cox proportional hazards model adjusted for baseline age and sex. Additional covariates selected based on a univariate Cox proportional hazards model (*P* < .10) included mean alcohol consumption (10 g/day), mean total energy intake (100 kcal/day), mean carbohydrate intake (% energy), mean plant-based protein intake (% energy), baseline duration of diabetes, HbA1c, systolic blood pressure, log triglycerides, uric acid, log urinary albumin excretion, sulfonylurea use, glucagon-like peptide-1 receptor agonist use, calcium channel blocker use, and antiplatelet agent use.

### Association between sleep duration and the incidence and progression of diabetic nephropathy

To further characterize the association between sleep duration and the composite endpoint, particularly in view of a potential nonlinear relationship, sleep duration was additionally analyzed as a categorical variable. Participants were categorized into 4 groups according to their mean sleep duration (<6, 6 to <7, 7 to <8, and ≥8 hours). The distribution of mean sleep duration in the study cohort is shown in Fig. S1 (26). One participant who experienced the composite endpoint was excluded from this analysis because no sleep duration measurements were available before the event, precluding calculation of the mean sleep duration.

At baseline, participants in the longer sleep duration groups tended to be older and predominantly male, and to have lower body mass index (BMI), lower LDL cholesterol levels, and lower eGFR, with HbA1c levels being the lowest in the 7 to <8 hours group. The prevalence of diabetic retinopathy was also higher in the longer sleep duration groups. In contrast, no significant differences were observed in blood pressure or UAE across sleep duration groups (Table 3).

**Table 3:** Comparison of participants’ demographic and baseline characteristics among 4 sleep duration groups

Mean sleep duration	<6 hours (n = 174)	6 to <7 hours (n = 273)	7 to <8 hours (n = 170)	≥8.0 hours (n = 60)	*P* for trend
Sex (male) (%)	92 (52.9)	170 (62.7)	112 (65.9)	45 (75.0)	.001
Age (years)	55.9 ± 8.7	57.4 ± 8.5	59.1 ± 8.5	62.0 ± 5.2	<.001
Body mass index (kg/m^2^)	25.3 ± 4.4	24.8 ± 4.1	23.8 ± 3.9	23.8 ± 3.4	<.001
Duration of diabetes (years)	8.0 (4.0 to 12.0)	8.0 (5.0 to 12.0)	10.0 (5.0 to 16.0)	8.0 (4.0 to 13.0)	.042
HbA1c (%)	7.02 ± 0.94	7.01 ± 1.01	6.74 ± 0.82	6.89 ± 1.01	.018
Systolic blood pressure (mmHg)	128.0 ± 14.2	125.5 ± 13.7	125.7 ± 14.5	127.4 ± 12.8	.407
Diastolic blood pressure (mmHg)	77.4 ± 10.3	76.5 ± 10.7	76.3 ± 9.8	76.6 ± 10.5	.437
Total cholesterol (mg/dL)	186.1 ± 26.5	184.7 ± 28.4	183.2 ± 28.2	185.8 ± 24.5	.566
Low-density lipoprotein cholesterol (mg/dL)	106.2 ± 25.0	104.4 ± 25.2	100.4 ± 23.5	101.3 ± 22.0	.028
High-density lipoprotein cholesterol (mg/dL)	58.4 ± 12.8	57.9 ± 13.3	61.3 ± 15.5	60.2 ± 13.5	.053
Triglycerides (mg/dL)	99.0 (70.0 to 149.0)	100.0 (70.0 to 157.0)	94.0 (65.5 to 138.5)	113.0 (76.0 to 161.0)	.832
Uric acid (mg/dL)	5.52 ± 1.34	5.38 ± 1.25	5.50 ± 1.18	5.59 ± 1.00	.725
eGFR (mL/min/1.73 m^2^)	79.9 ± 17.9	79.4 ± 17.5	77.0 ± 16.2	71.8 ± 17.9	.002
UAE (mg/g creatinine)	10.0 (5.3 to 21.2)	10.0 (5.5 to 21.2)	9.9 (6.2 to 17.4)	9.7 (5.6 to 22.7)	.755
Presence of diabetic retinopathy	50 (28.7)	92 (34.1)	66 (39.3)	28 (46.7)	.005
Use of glucose-lowering agents					
Metformin	83 (47.7)	140 (51.7)	90 (52.9)	26 (43.3)	.950
Sulfonylurea	18 (10.3)	18 (6.6)	14 (8.2)	7 (11.7)	.975
Glinides	5 (2.9)	4 (1.5)	2 (1.2)	0 (0.0)	.111
Dipeptidyl peptidase-4 inhibitors	56 (32.2)	88 (32.5)	64 (37.6)	18 (30.0)	.645
Sodium-glucose cotransporter 2 Inhibitors	0 (0)	0 (0)	0 (0)	0 (0)	—
Thiazolidinediones	12 (6.9)	8 (3.0)	6 (3.5)	2 (3.3)	.153
α-Glucosidase inhibitors	92 (52.9)	149 (55.0)	100 (58.8)	33 (55.0)	.410
Glucagon-like peptide-1 receptor agonists	13 (7.5)	16 (5.9)	11 (6.5)	5 (8.3)	.964
Insulin	12 (6.9)	28 (10.3)	19 (11.2)	7 (11.7)	.172
Use of other drugs					
Angiotensin-converting enzyme inhibitors	0 (0.0)	2 (0.7)	2 (1.2)	0 (0.0)	.476
Angiotensin II receptor blockers	71 (40.8)	115 (42.4)	72 (42.4)	28 (46.7)	.504
Calcium channel blockers	34 (19.5)	62 (22.9)	46 (27.1)	9 (15.0)	.682
Statins	55 (31.6)	111 (41.0)	73 (42.9)	27 (45.0)	.024
Antiplatelet agents	6(3.4)	6(2.2)	7(4.1)	3(5.0)	.446
MEQ (points)	55.7 ± 6.5	57.4 ± 7.4	58.8 ± 6.8	58.7 ± 8.7	<.001
Pittsburgh Sleep Quality Index (points)	7.0 ± 3.5	5.1 ± 2.6	3.8 ± 2.1	3.1 ± 2.0	<.001
Sleep duration (h)	5.2 ± 0.8	6.3 ± 0.5	7.3 ± 0.5	8.4 ± 0.8	<.001
Beck Depression Inventory-II (points)	9.9 ± 7.4	9.5 ± 7.1	9.7 ± 7.6	10.2 ± 9.2	.928
Physical activity (Mets/h/wk)	43.2 ± 75.3	37.0 ± 60.9	48.2 ± 78.4	56.0 ± 103.6	.170
Current smoker (yes)	30 (17.2)	73 (26.7)	34 (20.0)	15 (25.0)	.422
Alcohol consumption (g/d)	0.2 (0.0, 10.9)	0.5 (0.0, 11.6)	0.5 (0.0, 25.5)	1.2 (0.0, 33.4)	<.001
Total energy intake (kcal/d)	1616.0 (1296.2 to 2085.7)	1664.6 (1324.0 to 1976.1)	1657.6 (1347.4 to 1987.8)	1687.5 (1368.1 to 2062.2)	.913
Carbohydrate intake (% energy)	52.5 ± 7.3	53.7 ± 7.7	52.6 ± 8.6	51.6 ± 10.0	.438
Fat intake (% energy)	26.7 ± 5.2	25.5 ± 5.0	25.1 ± 5.5	23.6 ± 6.2	<.001
Protein intake (% energy)	15.9 ± 2.7	15.4 ± 2.9	15.7 ± 3.3	15.3 ± 3.6	.571
Animal-based protein (% energy)	9.2 ± 2.7	8.7 ± 2.9	9.0 ± 3.4	8.8 ± 3.4	.571
Plant-based protein (% energy)	6.7 ± 1.1	6.7 ± 1.1	6.7 ± 1.1	6.4 ± 1.2	.290
Salt intake (g/day)	10.8 ± 3.5	10.9 ± 3.3	11.1 ± 4.0	10.8 ± 3.9	.607

Abbreviations: eGFR, estimated glomerular filtration rate; MEQ, Morningness–Eveningness Questionnaire; UAE, urinary albumin excretion

Data are presented as numbers (%), means ± standard deviations, or medians (quartiles 1-3). Except for variables presented as mean values, all variables represent baseline measurements. Trend estimation for linear trends across groups was based on linear regression analysis for continuous variables and logistic regression analysis for categorical variables.

Participants in the longer sleep duration groups were more likely to use statins, while no significant differences were observed in the use of glucose-lowering agents, antihypertensive agents, or antiplatelet agents across groups.

Regarding lifestyle habits, participants with longer sleep duration had significantly higher MEQ scores and lower PSQI scores, indicating greater morning preference and better subjective sleep quality. Alcohol consumption increased, and fat intake decreased with increasing sleep duration, whereas no significant trend was observed in total energy intake across sleep duration groups.

During follow-up, BMI remained consistently lower in participants with longer sleep duration, with significant differences observed between sleep duration groups at several time points (Table S3) (26). Similarly, HbA1c levels tended to be lower in the longer sleep duration groups, whereas participants with shorter sleep duration showed relatively higher HbA1c values over time (data not shown). On the other hand, longitudinal trajectories of both systolic and diastolic blood pressure were broadly similar across sleep duration groups throughout follow-up, with no significant trend differences observed at any time point (Tables S4 and S5) (26).

Estimated glomerular filtration rate was consistently lower over time among participants with longer sleep duration, particularly in the ≥8 hours group (Table S6) (26). Although no significant differences in log UAE were observed between groups at baseline, participants with longer sleep duration tended to exhibit higher log UAE levels over time (Table S7) (26). Furthermore, changes in log UAE from baseline were greater among those with longer sleep duration during follow-up (Table S8) (26).

Based on these observations, we next evaluated the association between sleep duration categories and the risk of the composite endpoint using multivariable Cox proportional hazards models. Compared with the reference group (<6.0 hours), the HRs were 1.08 (95% CI, 0.74-1.57) for 6 to <7 hours, 1.56 (95% CI, 1.05-2.31) for 7 to <8 hours, and 1.92 (95% CI, 1.15-3.19) for ≥8 hours, with a significant increasing trend (*P* for trend = .003) (Table 4). The results were consistent when sleep duration was reclassified into 3 categories: <6.0 hours, 6 to <7 hours, and ≥7 hours (Table S9) (26).

**Table 4:** Associations between sleep duration categories with the composite endpoint in multivariable Cox proportional hazards model Cox proportional hazards models

Parameters	Cases (%)	HR (95% CI)	*P* value for group comparison	*P* for trend
<6.0 hours	49(28.2)	1.00 (Reference)	.018	.003
6 to <7 hours	76(27.8)	1.08 (0.74 to 1.57)		
7 to <8 hours	63(37.1)	1.56 (1.05 to 2.31)		
≥8.0 hours	26(43.3)	1.92 (1.15 to 3.19)		

Cox proportional hazards model adjusted for baseline age and sex. Additional covariates selected based on a univariate Cox proportional hazards model (*P* < .10) included mean alcohol consumption (10 g/day), mean total energy intake (100 kcal/day), mean carbohydrate intake (% energy), mean plant-based protein intake (% energy), baseline duration of diabetes, HbA1c, systolic blood pressure, log triglycerides, uric acid, log urinary albumin excretion, sulfonylurea use, glucagon-like peptide-1 receptor agonist use, calcium channel blocker use, and antiplatelet agent use. *P* for trend was calculated by modeling sleep duration categories as an ordinal variable.

### Association between subgroups and the composite endpoint of the incidence and progression of diabetic nephropathy

To examine whether the association between sleep duration and the composite endpoint varied across key clinical subgroups, analyses were performed according to baseline albuminuria status, eGFR, and age.

In participants with normoalbuminuria, longer sleep duration was not significantly associated with the endpoint, although a modest trend was observed (Table S10) (26). In participants with microalbuminuria, longer sleep duration was associated with a substantially higher risk, and a significant interaction with microalbuminuria status was observed (*P* for interaction < .001).

In subgroup analyses stratified by baseline eGFR, longer sleep duration was associated with a higher risk of the endpoint among participants with eGFR <60 mL/min/1.73 m^2^, whereas the association was weaker among those with eGFR ≥60 mL/min/1.73 m^2^. No statistically significant interaction was observed (*P* for interaction = .753) (Table S11) (26).

In subgroup analyses stratified by age, a graded association between longer sleep duration and risk of the endpoint was observed among participants aged ≥65 years, whereas no significant association was observed in participants aged <65 years. A significant interaction with age category was identified (*P* for interaction = .046) (Table S12) (26).

## Discussion

This prospective cohort study of Japanese participants with type 2 diabetes demonstrated that longer habitual sleep duration was associated with an increased risk of the incidence and progression of DN. Participants reporting 7 to <8 hours or ≥8 hours of sleep had a higher risk than those reporting <6 hours of sleep (reference category). Subgroup analyses further revealed that this association was particularly pronounced among participants aged ≥65 years and those with microalbuminuria or reduced kidney function at baseline. These findings suggest that, even among participants with relatively favorable metabolic and lifestyle profiles, longer sleep duration may identify a subgroup at elevated risk of DN, particularly in older participants and those with microalbuminuria or reduced kidney function at baseline.

Previous studies on sleep duration and DN have been limited. A prior prospective study of 2959 people with type 2 diabetes followed for 2.3 years reported an association between long sleep duration and incidence of diabetic kidney disease (DKD) (30). Similarly, a recent large prospective cohort study in the United Kingdom, including 24 081 people with type 2 diabetes, found that both short and long sleep duration were independently associated with DKD development (31). In contrast to the latter study, shorter sleep duration was not significantly associated with DN incidence or progression in the present study. This discrepancy may reflect differences in study design, population characteristics, or outcome definitions. Our study extends the existing literature through several methodological strengths. First, habitual sleep duration was assessed using the mean of multiple repeated measurements rather than a single baseline assessment. Second, DN incidence and progression were defined using laboratory-based clinical criteria rather than administrative diagnostic codes. Third, we comprehensively adjusted for a wide range of lifestyle factors, including diet, physical activity, alcohol consumption, and other health-related behaviors. Finally, we evaluated longitudinal changes in metabolic risk factors and lifestyle behaviors across sleep duration groups.

An important distinction between previous studies (30, 31) and the present study is the metabolic and demographic profile of longer sleepers. In previous studies, people with longer sleep duration generally had higher BMI and adverse metabolic profiles, supporting a metabolically driven pathway. In contrast, in our study, participants with longer sleep duration had lower BMI and lower LDL cholesterol levels, with HbA1c levels being lowest in the 7 to <8 hours group. This difference suggests that, particularly among older participants, longer sleep duration may reflect underlying physiological vulnerability rather than metabolic excess. Among participants aged ≥65 years, longer sleep duration was associated with an increased risk of DN incidence and progression. However, because BMI did not differ materially across sleep duration groups among participants aged ≥65 years (data not shown), the observed association is unlikely to be explained by BMI and may instead reflect age-related physiological vulnerability. Prolonged sleep may reflect a compensatory response to disrupted sleep homeostasis, potentially indicating underlying physiological vulnerability rather than poor subjective sleep quality. Indeed, in our study, PSQI-assessed sleep quality was not independently associated with the endpoint, and participants with longer sleep generally reported better subjective sleep quality. This apparent paradox may reflect limitations of subjective sleep quality measures, which may not capture sleep architecture abnormalities or unrecognized sleep disorders. This finding should therefore be interpreted cautiously and warrants further investigation. Longer sleep duration may also influence protein metabolism and body composition, contributing to sarcopenia, falls, and fractures (32), and has been linked to elevated inflammatory markers (33, 34), which may contribute to the incidence and progression of DN. Overall, longer sleep duration may primarily reflect underlying systemic vulnerability, including frailty, reduced physical activity, and low-grade inflammation, rather than a direct causal effect.

Baseline albuminuria status modified the association between sleep duration and renal risk. Although longer sleep duration was not independently associated with the composite endpoint within the normoalbuminuria or microalbuminuria subgroups, a significant interaction was observed, suggesting heterogeneity by baseline kidney damage. Similarly, the association between longer sleep duration and DN risk was also observed among participants with an eGFR <60 mL/min/1.73 m^2^. Given the presence of reduced eGFR or microalbuminuria, these participants likely represented a population with underlying chronic kidney disease. Such people may have reduced physiological reserve and heightened susceptibility to underlying vulnerabilities, including frailty and chronic inflammation, which have been reported to be more prevalent among people with long sleep duration (33, 34). These observations, together with the lack of a consistent association with metabolic risk profiles and the absence of an independent relationship with sleep quality in the present study, suggest that longer sleep duration is more likely to reflect underlying systemic conditions such as frailty and chronic low-grade inflammation. Taken together, these findings suggest that longer sleep duration may serve as a clinically accessible marker of underlying physiological vulnerability, rather than a direct causal factor, for the incidence and progression of DN.

Several limitations should be noted. First, the observational cohort design made it impossible to determine the cause-and-effect relationships and pathological mechanisms. Second, one potential explanation for the relatively higher incidence of DN observed in this study compared with a previous study (35) is that DN was diagnosed based on a single measurement of random urine albumin concentration at each annual visit, whereas current guidelines recommend confirmation using repeated UAE measurements over a 3- to 6-month period (36). Given the substantial intra-individual variability in UAE, this approach may have resulted in misclassification and overestimation of DN incidence and progression. Such measurement variability may also have introduced misclassification bias, which should be considered when interpreting the magnitude of the observed associations. However, our sensitivity analyses support the robustness of the main findings. Specifically, participants in the longer sleep duration groups exhibited greater annual increases in UAE levels (Table S8) (26), despite albumin being assessed only once per year. This consistent directionality suggests that the observed association is unlikely to be solely attributable to measurement variability. Third, lifestyle habits were self-reported, which may introduce recall or social desirability bias. Fourth, although mean sleep duration was used to reflect long-term habitual exposure, within-subject variability in sleep duration over time may also influence renal outcomes. In supplementary analyses, sleep duration variability was generally modest across groups (Table S13) (26). Nevertheless, more detailed assessments of longitudinal sleep pattern instability were not available, and residual confounding related to unmeasured sleep variability cannot be fully excluded. Although additional analyses adjusting for baseline physician-diagnosed sleep apnea syndrome (Table S14) (26) and baseline sleep medication use (Table S15) (26) yielded essentially unchanged results, our database did not distinguish obstructive from central sleep apnea, nor did it include information on physician-diagnosed insomnia. Furthermore, other dimensions of sleep health, such as sleep timing and objectively assessed sleep quality, were unavailable. Therefore, residual confounding by unmeasured sleep characteristics cannot be excluded. Fifth, the number of events in several subgroups was limited, resulting in wide confidence intervals and reduced statistical precision, although subgroup analyses suggested associations among participants with microalbuminuria, reduced eGFR, and older age. Therefore, these findings should be interpreted cautiously and considered exploratory. Sixth, when considering renoprotective therapies, additional adjustment for baseline use of angiotensin-converting enzyme inhibitors (ACEI) or angiotensin receptor blockers (ARB) in the main multivariable model did not materially alter the significant association between longer sleep duration categories and the risk of the composite endpoint (data not shown). Although baseline use of GLP-1 receptor agonists was included in the multivariable analyses, detailed longitudinal changes in renoprotective therapies during follow-up, including initiation or modification of SGLT2 inhibitors, ACEIs/ARBs, and newer mineralocorticoid receptor antagonists, were not comprehensively captured. Notably, none of the participants were receiving SGLT2 inhibitors at baseline, likely reflecting the limited availability and early clinical adoption of these agents in Japan during the enrollment period (2013-2014). Similarly, GLP-1 receptor agonist use was uncommon at baseline, reflecting the limited use of earlier-generation agents available in Japan at that time. Because these medications may influence renal outcomes, residual confounding related to evolving treatment patterns cannot be fully excluded. Finally, although mean sleep duration did not reach conventional statistical significance in the initial multivariable model when analyzed as a continuous variable (*P* = .082), subsequent categorical analyses were performed to further explore a potential nonlinear association with renal outcomes. Because multiple subgroup, sensitivity, and categorical analyses were conducted without formal correction for multiple testing, the possibility of type I error or spurious findings cannot be fully excluded. Therefore, these secondary analyses should be interpreted cautiously, although the consistency of observed trends across categorical models supports the potential robustness of the overall association.

In conclusion, longer habitual sleep duration was associated with an increased risk of DN incidence and progression in participants with type 2 diabetes, particularly among older participants and those with microalbuminuria or reduced kidney function at baseline. Longer, but not shorter, sleep duration may serve as a readily available clinical marker of underlying vulnerability, even in individuals with otherwise favorable metabolic and lifestyle profiles.

## Data Availability

All data generated or analyzed during this study are included in this article or its supplementary material files. Further inquiries can be directed to the corresponding author.

## Clinical trial information

**Trial Registration:** University Hospital Medical Information Network Clinical Trials Registry: UMIN000010932

## Abbreviations

BDHQ: Brief Self-Administered Diet History Questionnaire
BMI: body mass index
CI: confidence intervals
DKD: diabetic kidney disease
DN: diabetic nephropathy
eGFR: estimated glomerular filtration rate
HRs: hazard ratios
MEQ: Morningness–Eveningness Questionnaire
PSQI: Pittsburgh Sleep Quality Index
UAE: urinary albumin excretion

## References

[1] Rout P, Jialal I. Diabetic Nephropathy. StatPearls; 2025. 30480939

[2] Oosterwijk MM, Groothof D, Navis G, et al High-normal protein intake is not associated with faster renal function deterioration in patients with type 2 diabetes: a prospective analysis in the DIALECT cohort. Diabetes care 2022;45:35-41. Diabetes Care. 2022;45(3):e69. 10.2337/dc21-1211 34711636

[3] Hayashino Y, Okamura S, Kurita N, et al Baseline renal function modified the association between total, plant or animal protein intake and the risk of developing renal composite outcome in people with type 2 diabetes: a prospective cohort study [diabetes distress and care registry at Tenri (DDCRT25)]. Acta Diabetol. 2025;62(3):375‐383. 10.1007/s00592-024-02364-4 39207491

[4] Kaji A, Hashimoto Y, Kobayashi Y, et al Protein intake is not associated with progression of diabetic kidney disease in patients without macroalbuminuria. Diabetes Metab Res Rev. 2019;35(5):e3150. 10.1002/dmrr.3150 30860658

[5] Dunkler D, Kohl M, Teo KK, et al Population-attributable fractions of modifiable lifestyle factors for CKD and mortality in individuals with type 2 diabetes: a cohort study. Am J Kidney Dis. 2016;68(1):29‐40. 10.1053/j.ajkd.2015.12.019 26830448

[6] Kelly JT, Su G, Zhang L, et al Modifiable lifestyle factors for primary prevention of CKD: a systematic review and meta-analysis. J Am Soc Nephrol. 2021;32(1):239‐253. 10.1681/ASN.2020030384 PMC7894668 32868398

[7] Chaput JP, Despres JP, Bouchard C, Tremblay A. The association between sleep duration and weight gain in adults: a 6-year prospective study from the Quebec Family Study. Sleep. 2008;31(4):517‐523. 10.1093/sleep/31.4.517 PMC2279744 18457239

[8] Yaggi HK, Araujo AB, McKinlay JB. Sleep duration as a risk factor for the development of type 2 diabetes. Diabetes Care. 2006;29(3):657‐661. 10.2337/diacare.29.03.06.dc05-0879 16505522

[9] Gottlieb DJ, Redline S, Nieto FJ, et al Association of usual sleep duration with hypertension: the Sleep Heart Health Study. Sleep. 2006;29(8):1009‐1014. 10.1093/sleep/29.8.1009 16944668

[10] Kim JH, Ha MJ, Choi DW, Park S. The impact of sleep duration on the incidence of new-onset chronic kidney disease in community-dwelling adults: a nationwide cohort study. Ann Med. 2025;57(1):2569986. 10.1080/07853890.2025.2569986 PMC12570226 41140193

[11] American Diabetes Association Professional Practice C . 5. Facilitating positive health behaviors and well-being to improve health outcomes: standards of care in diabetes-2025. Diabetes Care. 2025;48(Supplement_1):S86‐S127. 10.2337/dc25-S005 PMC11635047 39651983

[12] Ohkuma T, Fujii H, Iwase M, et al Association between sleep duration and urinary albumin excretion in patients with type 2 diabetes: the Fukuoka diabetes registry. PLoS One. 2013;8(11):e78968. 10.1371/journal.pone.0078968 PMC3827127 24265736

[13] Tan NYQ, Chan J, Cheng CY, Wong TY, Sabanayagam C. Sleep duration and diabetic kidney disease. Front Endocrinol (Lausanne). 2018;9:808. 10.3389/fendo.2018.00808 PMC6340267 30692966

[14] Kadoya M, Morimoto A, Miyoshi A, et al Sleep quality, autonomic dysfunction and renal function in diabetic patients with pre-CKD phase. Sci Rep. 2021;11(1):19048. 10.1038/s41598-021-98505-8 PMC8463568 34561498

[15] Osonoi Y, Mita T, Osonoi T, et al Morningness-eveningness questionnaire score and metabolic parameters in patients with type 2 diabetes mellitus. Chronobiol Int. 2014;31(9):1017‐1023. 10.3109/07420528.2014.943843 25102425

[16] Tokoro MF, Mita T, Osonoi Y, et al An evening chronotype is associated with the incidence and progression of diabetic retinopathy in people with type 2 diabetes mellitus: a cohort study. Diabetologia. 2026;69(2):504‐514. 10.1007/s00125-025-06590-5 41134330

[17] Mita T, Osonoi Y, Someya Y, et al Relationship of carbohydrate intake proportion to cardiovascular events in Japanese people with type 2 diabetes mellitus. J Clin Endocrinol Metab. 2025;110(11):e3587‐e3595. 10.1210/clinem/dgaf179 40114241

[18] Yamada T, Komatsu M, Komiya I, et al Development, progression, and regression of microalbuminuria in Japanese patients with type 2 diabetes under tight glycemic and blood pressure control: the Kashiwa study. Diabetes Care. 2005;28(11):2733‐2738. 10.2337/diacare.28.11.2733 16249548

[19] Agency SaT . Standard Tables of Food Composition in Japan (in Japanese). 5th rev ed. Printing Bureau of the Ministry of Finance; 2005.

[20] Kobayashi S, Honda S, Murakami K, et al Both comprehensive and brief self-administered diet history questionnaires satisfactorily rank nutrient intakes in Japanese adults. J Epidemiol. 2012;22(2):151‐159. 10.2188/jea.JE20110075 PMC3798594 22343326

[21] Hone JA, Ostberg O. A self-assessment questionnaire to determine morningness-eveningness in human circadian rhythms. Int J Chronobiol. 1976;4:97‐110. 1027738

[22] Buysse DJ, Reynolds CF, 3rd, Monk TH, Berman SR, Kupfer DJ. The Pittsburgh Sleep Quality Index: a new instrument for psychiatric practice and research. Psychiatry Res. 1989;28(2):193‐213. 10.1016/0165-1781(89)90047-4 2748771

[23] Beck AT, Steer RA, Brown GK. Manual for the Beck Depression Inventory-II. Psychological Corporation; 1996.

[24] Craig CL, Marshall AL, Sjostrom M, et al International physical activity questionnaire: 12-country reliability and validity. Med Sci Sports Exerc. 2003;35(8):1381‐1395. 10.1249/01.MSS.0000078924.61453.FB 12900694

[25] Matsuo S, Imai E, Horio M, et al Revised equations for estimated GFR from serum creatinine in Japan. Am J Kidney Dis. 2009;53(6):982‐992. 10.1053/j.ajkd.2008.12.034 19339088

[26] Ikeda F, Mita T, Osonoi Y, et al Data from: Supplementary materials. Longer sleep duration and the incidence and progression of diabetic nephropathy: A prospective cohort study. *Figshare*. 2026. 10.6084/m9.figshare.32407353

[27] Katano S, Nakamura Y, Nakamura A, et al Association of short sleep duration with impaired glucose tolerance or diabetes mellitus. J Diabetes Investig. 2011;2(5):366‐372. 10.1111/j.2040-1124.2011.00114.x PMC4019304 24843515

[28] Hall MH, Muldoon MF, Jennings JR, Buysse DJ, Flory JD, Manuck SB. Self-reported sleep duration is associated with the metabolic syndrome in midlife adults. Sleep. 2008;31(5):635‐643. 10.1093/sleep/31.5.635 PMC2398755 18517034

[29] Hwang I, Oh D, Yoon JE, et al Association between sleep duration and thirst in a nationally representative cross-sectional survey. Sci Rep. 2026;16(1):22306. 10.1038/s41598-026-53338-1 PMC13377167 42143124

[30] Liu C, Zhang J, Wei X, et al Effects of sleep duration and changes in body mass index on diabetic kidney disease: a prospective cohort study. Front Endocrinol (Lausanne). 2023;14:1278665. 10.3389/fendo.2023.1278665 PMC10641014 37964958

[31] Niu X, Liu H, Wang Y, et al Sleep duration, mediating biomarkers, and risk of microvascular complications among individuals with type 2 diabetes: a prospective cohort study. Diabetes Res Clin Pract. 2025;221:112026. 10.1016/j.diabres.2025.112026 39929336

[32] Piovezan RD, Abucham J, Dos Santos RV, Mello MT, Tufik S, Poyares D. The impact of sleep on age-related sarcopenia: possible connections and clinical implications. Ageing Res Rev. 2015;23(Pt B):210‐220. 10.1016/j.arr.2015.07.003 26216211

[33] Lee WJ, Peng LN, Liang CK, Chiou ST, Chen LK. Long sleep duration, independent of frailty and chronic inflammation, was associated with higher mortality: a national population-based study. Geriatr Gerontol Int. 2017;17(10):1481‐1487. 10.1111/ggi.12899 27679974

[34] Barutta F, Bruno G, Grimaldi S, Gruden G. Inflammation in diabetic nephropathy: moving toward clinical biomarkers and targets for treatment. Endocrine. 2015;48(3):730‐742. 10.1007/s12020-014-0437-1 25273317

[35] Katayama S, Moriya T, Tanaka S, et al Low transition rate from normo- and low microalbuminuria to proteinuria in Japanese type 2 diabetic individuals: the Japan Diabetes Complications Study (JDCS). Diabetologia. 2011;54(5):1025‐1031. 10.1007/s00125-010-2025-0 PMC3071947 21286682

[36] American Diabetes Association Professional Practice Committee for Diabetes . 11. Chronic kidney disease and risk management: standards of care in diabetes-2026. Diabetes Care. 2026;49(Supplement_1):S246‐s260. 10.2337/dc26-S011 PMC12690176 41358881

